# The Association of Loneliness and Social Isolation with Survival in Cancer Patients: A Systematic Review and Meta-Analysis

**DOI:** 10.3390/epidemiologia7050135

**Published:** 2026-09-28

**Authors:** Irena Ilic, Milena Ilic

**Affiliations:** 1 Faculty of Medicine, University of Belgrade, 11000 Belgrade, Serbia; 2 Department of Epidemiology, Faculty of Medical Sciences, University of Kragujevac, 34000 Kragujevac, Serbia

**Keywords:** cancer, loneliness, social isolation, mortality, cancer-specific mortality, systematic review, meta-analysis

## Abstract

Background/Objectives: Social isolation and loneliness are distinct but related dimensions of social disconnection that may influence outcomes among individuals with cancer. Previous reviews frequently combined heterogeneous social constructs and often did not distinguish assessments conducted before and after cancer diagnosis. Methods: A systematic review and meta-analysis were conducted following registration in PROSPERO (CRD420261359008). PubMed, Scopus and Web of Science were searched from inception to 2 April 2026. Studies evaluating loneliness and/or social isolation after cancer diagnosis and reporting all-cause or cancer-specific mortality were eligible. Random-effects meta-analyses were performed. Pooled results are reported as hazard ratios (HRs) with 95% confidence intervals (CIs). Results: In total, nine studies involving 20,367 patients with cancer were included. Higher loneliness was associated with increased all-cause mortality (HR = 1.79, 95% CI 1.39–2.31; I^2^ = 16%). Assessments of social isolation and overall mortality demonstrated a significant association with increased risk of death (HR = 1.36, 95% CI 1.18–1.56; I^2^ = 22%). A significant association between higher levels of social isolation and cancer-specific mortality (HR = 1.21, 95% CI 1.03–1.42; I^2^ = 8%) was shown. Sensitivity analyses generally supported the robustness of the findings; however, the association between social isolation and cancer-specific mortality became non-significant after exclusion of the largest contributing cohort. Conclusions: Higher levels of loneliness and social isolation assessed after cancer diagnosis were associated with poorer survival outcomes among patients with cancer. These findings support the importance of considering social disconnection during cancer survivorship and treatment. However, the small number of included studies limits the strengths of conclusions. Further large prospective studies with repeated assessments are needed to clarify these associations and their potential implications for supportive cancer care.

## 1. Introduction

Advances in cancer detection and treatment have substantially improved survival worldwide. In 2022, an estimated 53.5 million people were alive within 5 years of a cancer diagnosis [1]. The global cancer burden is projected to increase further [2]. Consequently, cancer care has increasingly expanded from an acute treatment model toward long-term survivorship management, emphasizing not only clinical and disease-related characteristics but also broader psychosocial and behavioral determinants that may influence outcomes [3]. Recently, social connection has been increasingly recognized as an important public health issue, supported by accumulating evidence linking social experiences with morbidity, quality of life and mortality across multiple health conditions [4,5]. The US Surgeon General’s Advisory on the “epidemic” of loneliness and isolation emphasizes the importance of social connection for health and well-being. According to the World Health Organization’s Commission on Social Connection, the global prevalence of loneliness in the 2014–2023 period was nearly 16% and the prevalence of social isolation 25–34%.

Among the social determinants of health and well-being, social isolation and loneliness have emerged as important yet conceptually distinct factors. Social isolation refers to the objective absence or limitation of social contacts, participation, roles or network structure, whereas loneliness is a subjective and distressing perception that one’s social relationships are insufficient in quantity or quality compared to one’s desired experience [4,5,6]. Although related as two forms of social disconnection [4], these constructs are not interchangeable and may occur independently, i.e., individuals may be socially connected yet feel lonely or socially isolated without experiencing loneliness [6]. Importantly, these terms also differ from concepts frequently used as proxy social measures in observational research, including marital status, living arrangement, household composition or perceived social support [7]. Although these measures may overlap, they do not fully capture the complex social perspectives of social isolation or loneliness.

Several mechanisms have been proposed to link social isolation and loneliness with cancer outcomes. Although these mechanisms may overlap, their relative importance may differ between the two constructs, and current evidence does not establish that they are identical. Biological pathways include dysregulation of stress-response systems, chronic inflammation, altered immune functioning, gene expression and neuroendocrine changes that may affect disease progression [8,9,10,11]. Behavioral mechanisms may additionally contribute through reduced treatment adherence, delayed healthcare utilization, lower engagement in healthy behaviors (e.g., lower physical activity, poorer nutrition, and sleep disruption), impaired symptom management and reduced access to supportive resources [12,13,14,15,16]. Psychological processes include a decreased sense of meaning and purpose, reduced resilience, difficulties coping with stressful situations and hopelessness [17,18,19]. Altogether, these diverse pathways provide mechanistic plausibility for a potential association between social disconnection and mortality among individuals with cancer. Potential overlap in these pathways does not negate the conceptual distinction between loneliness and social isolation or the rationale for examining them separately.

Nevertheless, the findings on the impact of social disconnection on cancer mortality from primary studies are not consistent. Differences in populations, exposure assessments, outcome definitions and follow-up durations have contributed to inconsistent findings across studies [20,21,22,23]. Additionally, previous reviews have often combined heterogeneous constructs under broad categories of social relationships or social factors, frequently combining objective social isolation with subjective loneliness [24] or relying on or including indirect proxies or distinct social factors, such as marital status or social support [24,25]. This conceptual overlap may obscure potentially distinct associations and complicate the interpretation of pooled estimates. Furthermore, variations in the timing of exposure assessments might lead to limited comparability across the literature. Notably, the currently available evidence appears to predominantly reflect assessments of social experiences among individuals prior to having their cancer diagnosis established [22,23,26,27]. Additionally, existing meta-analyses do not differentiate the timing of measurements, which might capture these social determinants as general baseline lifestyle risk factors only, without taking into account the critical shifts in dynamics of social connections that often occur after cancer diagnosis [28].

The aim of this systematic review and meta-analysis was to synthesize available evidence on the association between post-diagnosis social isolation and loneliness among individuals with cancer and overall and cancer-specific mortality.

## 2. Materials and Methods

This systematic review with a meta-analysis was conducted following the guidelines of the Preferred Reporting Items for Systematic Reviews and Meta-Analyses (PRISMA) 2020 (Supplementary Table S1) [29]. The research protocol was registered in the Prospective Register of Systematic Reviews (PROSPERO, CRD420261359008). The aim of this research was to assess the association between loneliness and social isolation with mortality outcomes in patients with cancer.

### 2.1. Literature Search

Searches were conducted in PubMed, SCOPUS and Web of Science (WOS). All databases were searched on 2 April 2026 by two researchers independently. There were no date or language restrictions applied, or any filters. The search strategies and terms employed across the databases are provided in Supplementary Table S2. The search strategy focused specifically on loneliness and social isolation as the exposures of interest, while terms such as social networks, social ties, social integration and social connectedness, which may be used to operationalize social isolation, were not included as standalone search concepts because they may also represent related but distinct constructs. The database search was supplemented by screening the reference lists of relevant reviews and included studies and articles that cited the included studies.

### 2.2. Eligibility Criteria

Studies were included if they involved adult patients (≥18 years) with cancer, in whom loneliness and/or social isolation were assessed using validated or non-validated instruments following their diagnosis. Studies were included if they reported an association between recorded loneliness and/or social isolation and all-cause mortality and/or cancer-specific mortality.

Study designs which were considered involved observational studies, i.e., cohort studies, registry-based studies and case-control studies. In cases of multiple reports of the same study population, the report containing the most data or the most recent data was included.

Studies were excluded if they involved patients < 18 years of age, if the participants did not have a confirmed diagnosis of cancer, if the study contained mixed populations where cancer-specific data could not be extracted separately, if the study did not assess loneliness and/or social isolation or assessed only related constructs without a clear measure of loneliness and/or social isolation, and where loneliness and/or social isolation were not possible to distinguish from other reported psychosocial constructs.

Studies that did not assess all-cause mortality and/or cancer-specific mortality and did not provide information on their association with the presence or levels of loneliness and/or social isolation were excluded, as well as studies without extractable effect estimates. Study types which were excluded were reviews, systematic reviews, meta-analyses, editorials, conference abstracts, letters, commentaries, and qualitative studies.

### 2.3. Selection and Assessment of Studies

After duplicates were removed, two researchers independently screened titles and abstracts. Next, full text articles were checked against inclusion and exclusion criteria. Any discrepancies were resolved by consensus.

Risk of bias and quality assessments of the included studies were done using the Newcastle–Ottawa scale (NOS) [30] independently by two reviewers, with any disagreements resolved through discussion. Each study could be awarded up to 9 stars across three categories—selection of participants, comparability of cohorts and ascertainment of outcome—with studies scoring ≥ 7 stars being considered as high quality.

### 2.4. Statistical Analysis

The DerSimonian and Laird generic inverse-variance random effects meta-analytical method was applied [31]. Hazard ratios (HRs) with corresponding 95% Confidence Intervals (95% CIs), or data necessary for computing the effect estimate, were extracted from primary studies by both authors independently, and any disagreements were resolved by consensus. Effect estimates labelled as relative risks were interpreted as HRs when derived from Cox proportional hazards regression models. When available, data from the most-adjusted multivariable models were always extracted. Supplementary Table S3 reports variables for which adjustments were made across all included studies. When studies reported separate effect estimates for distinct cancer-site participant groups or analytic cohorts, one outcome per group or cohort was included in the relevant outcome-specific meta-analysis and treated as statistically independent.

When estimating the risk, the least lonely and the most socially connected group was selected as the reference group, while the comparison group was the loneliest or the least socially connected group. In order to ensure consistency of reference, all effect estimates from primary studies, where necessary, were converted to match this, by inverting as follows, 1/effect estimate, 1/upper 95% CI bound, and 1/lower 95% CI bound, i.e., as reciprocal values of the original values [32,33]. When studies reported effect estimates across different levels of exposure (loneliness and/or social isolation), the highest vs. lowest comparison was extracted [34]. In one study, the highest exposure category included only 5 participants; therefore, the estimate for the next-highest exposure category was used because the estimate for the extreme category was considered potentially unstable. A sensitivity analysis excluding this study was conducted to assess the influence of this decision. While the direction of the exposure contrasts was harmonized, the thresholds used to define the comparison groups differed across studies. Therefore, the pooled estimates represent higher versus lower levels of loneliness or social isolation, rather than effects based on a common exposure threshold.

Statistical heterogeneity was assessed using the I^2^ statistic, whose values were interpreted as 0–40% might not be important, 30–60% might represent moderate heterogeneity, 50–90% might represent substantial heterogeneity, and 75–100% represents considerable heterogeneity [32]. In accordance with data availability, subgroup analyses were planned based on the use of validated vs. non-validated instruments and cancer types.

Sensitivity analyses were planned to examine the robustness of results by excluding studies with NOS scores < 7 and those using non-validated exposure measures and by the leave-one-out method. Additionally, a sensitivity analysis was conducted by excluding one study where the third instead of the fourth quartile of social isolation was used as the reference group due to the fourth quartile comprising only 5 patients.

The results of the meta-analysis are presented graphically using forest plots, with the pooled summary result presented as a diamond at the bottom and its width representing the confidence interval of the pooled estimate.

Depending on the number of included studies, i.e., if *n* ≥ 10, visual inspection of funnel plots was planned for an assessment of publication bias. All analyses were conducted in Review Manager (RevMan) version 5.4.1 [35].

## 3. Results

The literature search identified 2192 articles across three databases. After duplicates were removed, 1631 records were screened, and the full text of 187 articles was reviewed. Finally, 9 studies were included (Figure 1).

### 3.1. Study Characteristics

The characteristics of the included studies are shown in Table 1. Across the 9 considered studies [36,37,38,39,40,41,42,43,44], sample sizes ranged from 204 to 9267, comprising a total of 20,367 patients with cancer across all studies. Some patient populations included all cancer types [37] or several cancer types [36,39,40], whereas some considered breast cancer [38,41,42], colorectal cancer [43], and lung cancer [44]. The majority of studies were conducted in the USA [37,38,39,40,42,43], with one comprising the USA and China [41] and one in Colombia [36] and Japan [44].

Loneliness and social isolation were estimated after cancer diagnosis using validated scales. The instrument used to estimate loneliness was the University of California, Los Angeles—UCLA—loneliness scale [36,37,44], while social isolation was estimated most often using the Berkman–Syme Social Network Index (SNI) [38,39,40,42,43], the After Breast Cancer Pooling Project—ABCPP—adaptation of the SNI [41] and the Luben Social Network Scale—LSNS-6 [44]. According to the NOS scale, 7 studies demonstrated a score of 7 or more, and 2 had a score of 6. Supplementary Table S3 provides details on variables used for adjustments in each study, the timing of exposure assessment relative to cancer diagnosis and the beginning of mortality follow-up.

### 3.2. Loneliness and Mortality in Patients with Cancer

There were 3 studies, totaling 3982 patients, which assessed the impact of post-diagnosis loneliness on mortality in patients with breast, prostate, cervical, gastrointestinal and lung cancer. Overall, higher loneliness was associated with increased all-cause mortality in patients with cancer (HR = 1.79, 95% CI 1.39–2.31, *p* < 0.00001) (Figure 2). Statistical heterogeneity was low (I^2^ = 16%; Chi^2^ = 2.37, *p* = 0.31), but this should be interpreted with caution due to the number of studies included in this analysis.

Sensitivity analyses indicated that the pooled estimates for the association between loneliness and mortality in cancer patients remained stable (Table 2). Restricting the loneliness analysis to studies reporting adjusted estimates did not alter the pooled result.

### 3.3. Social Isolation and Mortality in Patients with Cancer

There were 6 studies which assessed the impact of social isolation estimated post-diagnosis on all-cause mortality in patients with cancer. These studies comprised 15,585 patients in total, with glioma, breast, pancreatic, gastric, lung, colorectal and prostate cancer. The pooled estimate showed that patients with cancer who had higher levels of social isolation had an increased risk of all-cause mortality (HR = 1.36, 95% CI 1.18–1.56, *p* < 0.0001) (Figure 3). Statistical heterogeneity was low, with I^2^ = 22% and Chi^2^ = 7.73, *p* = 0.26.

Five studies assessed the impact of social isolation on cancer-specific mortality. In total, these studies comprised 16,181 patients suffering from breast, colorectal and prostate cancer. The risk for cancer-specific mortality was higher in patients with cancer who had higher levels of social isolation (HR = 1.21, 95% CI 1.03–1.42) (Figure 4). Statistical heterogeneity was low, with I^2^ = 8% and Chi^2^ = 7.63, *p* = 0.37.

Excluding two studies with lower NOS scores for quality did not affect the pooled estimates for any outcome. Leave-one-out sensitivity analyses demonstrated that exclusion of any single study did not substantially affect the pooled HR (range 1.32–1.44) for the association of overall survival with social isolation in cancer patients, which remained statistically significant. For cancer-specific survival, exclusion of the Kroenke LACE/NHS/SBCSS cohort (which contributed the largest weight—38.5%) [41] attenuated the pooled HR to 1.08 and rendered the association non-significant. Exclusion of all other studies did not substantially alter the findings (Table 2).

## 4. Discussion

This systematic review and meta-analysis synthesized evidence on post-diagnosis loneliness and social isolation as distinct constructs in relation to mortality among individuals with cancer. Higher levels of loneliness were associated with increased all-cause mortality, while higher levels of social isolation were associated with both higher all-cause and cancer-specific mortality. Statistical heterogeneity was low across analyses, and sensitivity analyses supported the robustness of the findings for all-cause mortality, whereas the association with cancer-specific mortality was sensitive to the exclusion of the largest cohort.

The results are broadly consistent with previous reviews reporting that social disconnection is associated with adverse outcomes among individuals with cancer. Pinquart et al. found that social networks were associated with mortality of cancer patients, with larger social networks reducing the relative risk of mortality by 20% (RR = 0.80, 95% CI 80–0.72) [25]. Similar to the present study, Cheng et al. found that social isolation and loneliness increased all-cause and cancer-specific mortality (HR = 1.34 and 1.11, respectively) [24]. However, earlier reviews frequently combined conceptually distinct constructs, including loneliness, social isolation, social support, marital status and living arrangements, making it difficult to determine whether specific dimensions of social relationships contribute differently to prognosis. Furthermore, most previous evidence has predominantly reflected social experiences assessed before cancer diagnosis or did not differentiate between before and after assessments. Consequently, less is known about whether social disconnection experienced during survivorship and treatment is similarly associated with outcomes.

The distinction between loneliness and social isolation is relevant because they represent subjective and objective dimensions of social disconnection, respectively, and may occur independently [4,5,6]. Although they may influence outcomes through overlapping behavioral and biological pathways [11,17,18], combining them may obscure construct-specific associations with mortality and make it difficult to determine whether their prognostic relevance differs. Examining them separately therefore allows for the available evidence for each construct to be interpreted more precisely. This approach is also consistent with evidence from general population and older-adult samples, where large systematic reviews and meta-analyses have demonstrated that both constructs are significantly associated with increased mortality [34,45,46,47].

The findings of the present review suggest that social disconnection assessed after cancer diagnosis may be associated with mortality outcomes, highlighting the importance of considering these experiences throughout the disease trajectory rather than solely before diagnosis. By focusing on post-diagnosis assessments, the present review aimed to reduce conceptual heterogeneity and better capture social experiences occurring after diagnosis, a period during which social relationships, support needs and health-related behaviors may substantially change [28].

Notably, in this meta-analysis the evidence for loneliness was derived from only three studies [36,37,44], two of which reported statistically significant associations with mortality [36,37]. Similarly, although social isolation showed significant pooled associations with both mortality outcomes, the precision of the pooled estimate for cancer-specific mortality was particularly influenced by a large cohort from the ABCPP study which contributed a substantial proportion of the overall weight [41]. The attenuation of the pooled estimate following exclusion of the largest contributing cohort suggests that the current evidence base for cancer-specific mortality remains limited and that additional large prospective studies are needed to improve estimate precision. Consequently, the available evidence for cancer-specific mortality, while suggestive of a meaningful association, should not yet be considered definitive.

Statistical heterogeneity was low across all analyses. However, this should not be interpreted as clinical or methodological homogeneity. Variations in the magnitude of associations across studies may reflect differences in included cancer populations, which ranged from site-specific cohorts, such as breast, colorectal, pancreatic and lung cancer, to studies involving multiple cancer types, as well as differences in disease stages, treatment modalities, follow-up durations and adjustment strategies. These differences should be considered when interpreting the pooled estimates and their generalizability. Additionally, previous evidence has suggested that associations between loneliness and particularly social isolation may vary across geographical regions [45], highlighting the need for studies from a broader range of socioeconomic and cultural settings.

The findings of this meta-analysis suggest that assessments of social disconnection may represent a potentially valuable component of supportive cancer care. Although the current evidence does not support causal inference or specific intervention recommendations, it highlights the importance of recognizing loneliness and social isolation as potentially relevant psychosocial factors during survivorship and treatment. A recent network meta-analysis showed that group logotherapy was the most effective non-pharmacological intervention for reducing loneliness and social isolation in patients with cancer, followed by psychoeducational therapy and supportive expressive group therapy [48]. However, Zhao et al. [48] highlighted the issue of scarcity of evidence, as was noted by previous systematic reviews [49,50]. Although some interventions may affect both constructs, the current evidence is insufficient to recommend specific construct-tailored interventions. Future intervention studies should clearly specify whether loneliness, social isolation or both are being targeted and should assess and report these outcomes separately. Further research into risk factors [51] and routine post-diagnosis screening for social disconnection may help guide supportive services. Such efforts could be supported by increasing awareness among healthcare professionals, patients and communities [11]; developing consensus on the timing and instruments used for the assessment [52]; establishing appropriate referral pathways; and identifying meaningful outcomes for following. Future research should also determine whether these associations vary across cancer populations and phases of care.

This study has several strengths. First, to the best of our knowledge this is among the first meta-analyses of the associations between social isolation and loneliness and mortality in a population of cancer patients, with these constructs examined separately and with a focus on post-diagnosis assessments. Next, the considered primary studies used validated instruments for assessing social isolation and loneliness. Additionally, low statistical between-study heterogeneity was observed across the pooled analyses.

Several limitations should be considered. First, the number of eligible studies was small, particularly for loneliness, limiting statistical power and precluding detailed subgroup analyses. Furthermore, the small number of studies introduces uncertainty in the estimation of between-study variance and confidence intervals in the random-effects analyses; therefore, the pooled estimates should be interpreted cautiously. Second, all included studies were observational, and residual confounding could not be excluded despite multivariable adjustment in most cohorts. Although the sensitivity analysis that was restricted to adjusted estimates yielded a similar pooled result, the pooled analysis included one unadjusted estimate and estimates based on different covariate adjustment sets. These differences may limit comparability across studies, as the study-specific estimates may not reflect precisely the same underlying association. Third, substantial variation existed in cancer type, disease severity, exposure categorization and covariate adjustment strategies. The timing of exposure assessments ranged from shortly after diagnosis to several years into survivorship, which may limit temporal comparability across studies and should be considered when interpreting the findings. Fourth, most evidence originated from high-income and upper-middle-income countries, potentially limiting generalizability across diverse healthcare systems and sociocultural contexts. Fifth, men were underrepresented, reflecting the inclusion of several large women-only cohorts. In addition, sex-specific estimates were not consistently reported by the primary studies, limiting the ability to assess whether associations differ by sex and limiting the generalizability of the findings to male patients.

In addition, although this review focused on post-diagnosis measurements to improve temporal relevance, repeated assessments of loneliness and social isolation over time were generally unavailable. Consequently, the dynamic nature of social relationships throughout the cancer trajectory could not be examined. Repeated assessments may be particularly informative, as research shows that levels of social isolation and loneliness change and increase with increasing time from diagnosis [53].

## 5. Conclusions

This meta-analysis suggests that higher levels of both loneliness and social isolation assessed after cancer diagnosis are associated with poorer survival outcomes among individuals with cancer. By examining these constructs separately and focusing on post-diagnosis assessments, this review adds to the emerging evidence that social experiences during the disease trajectory may be relevant to patient outcomes. Nevertheless, the limited number of studies, in particular for loneliness and cancer-specific mortality, indicates that further prospective research is needed. Future studies should incorporate repeated assessments of loneliness and social isolation, distinguish clearly between these constructs and determine whether changes in social disconnection over time influence cancer outcomes and represent modifiable targets for supportive care.

## Figures and Tables

**Figure 1:**
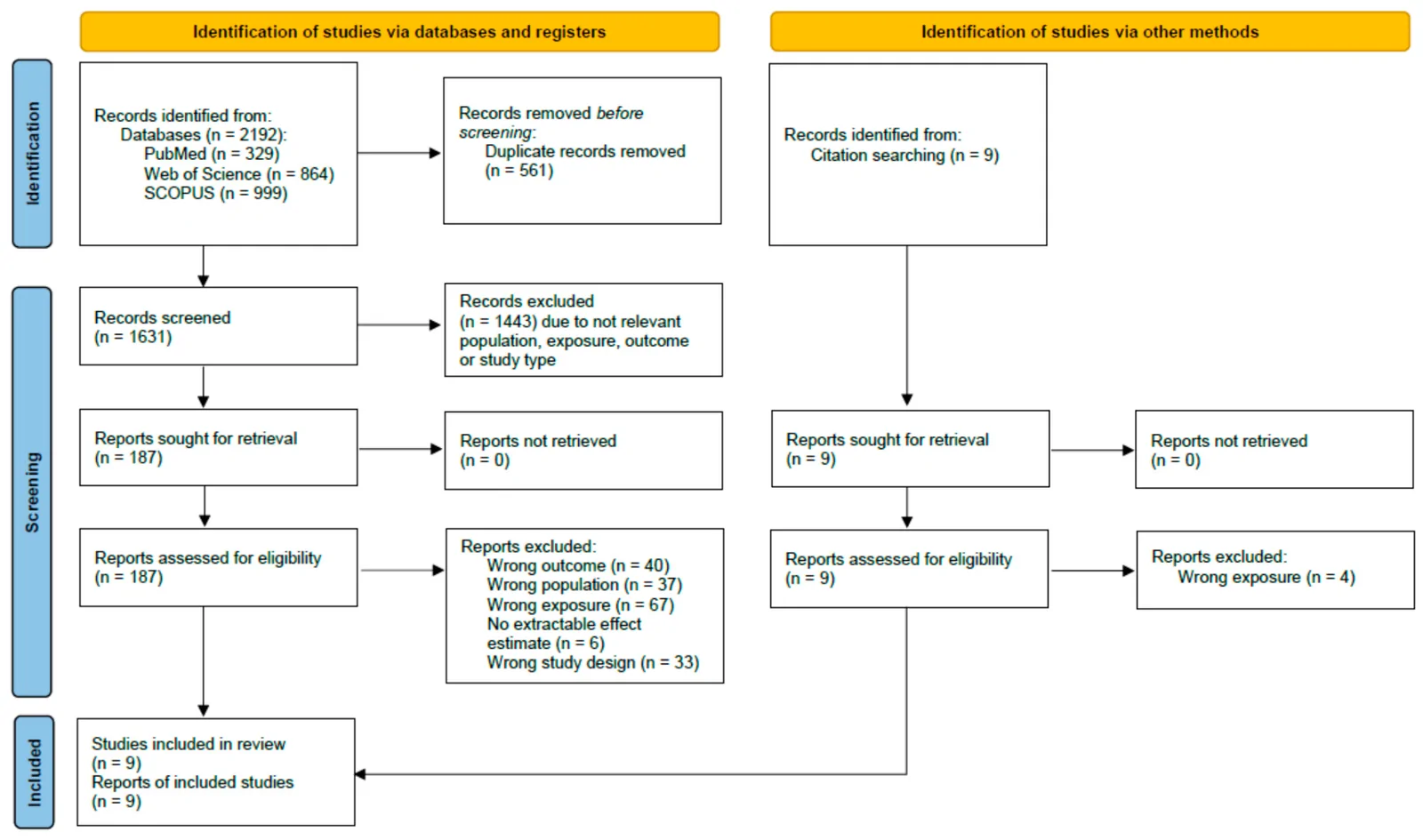
Flow diagram of literature search.

**Figure 2:**
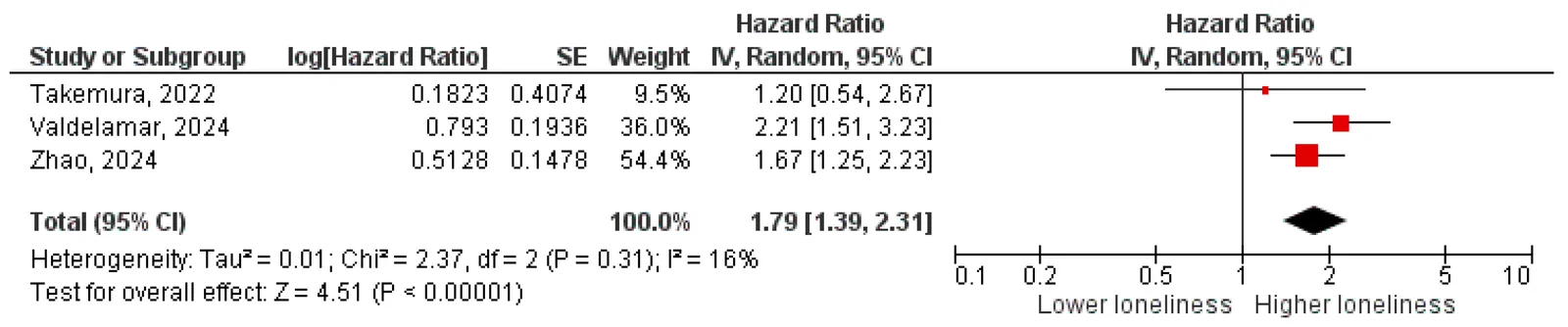
Effect of loneliness on overall mortality in patients with cancer [36,37,44].

**Figure 3:**
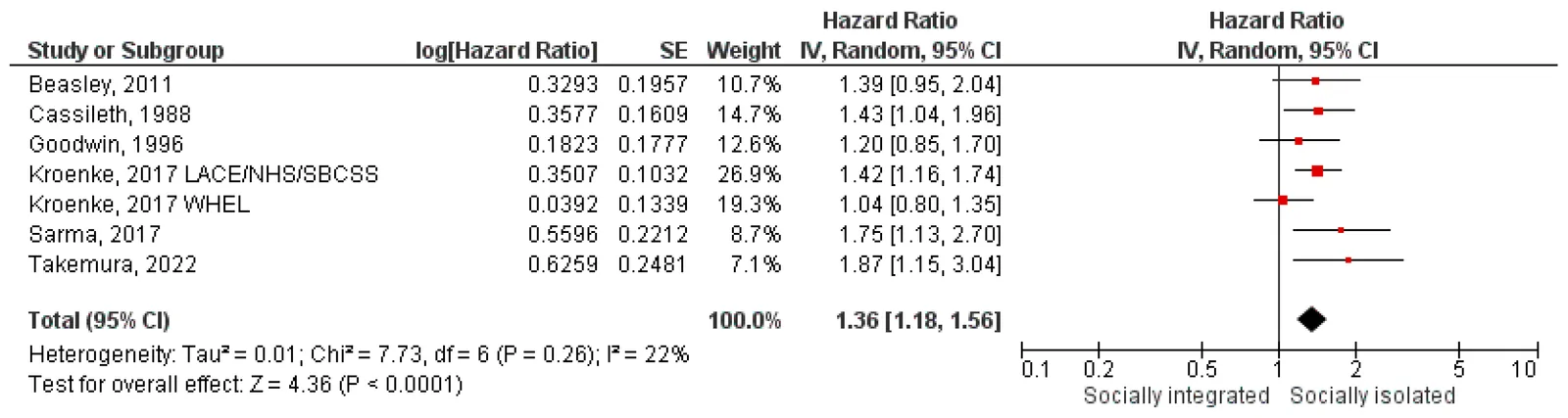
Effect of social isolation on overall mortality in patients with cancer [38,39,40,41,43,44].

**Figure 4:**
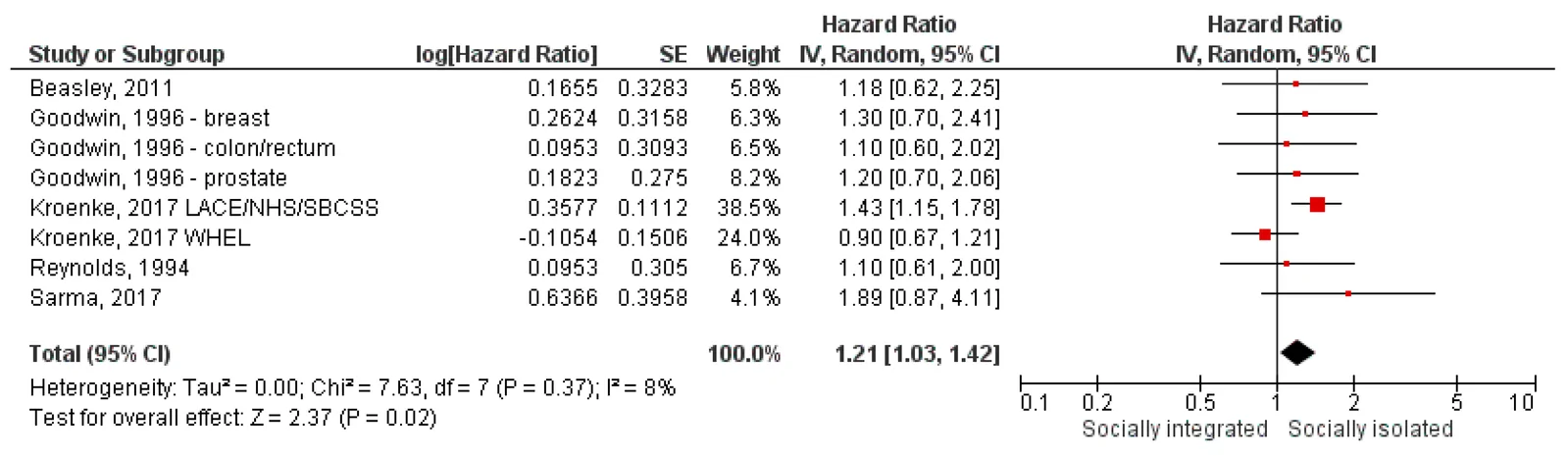
Effect of social isolation on cancer-specific mortality in patients with cancer [38,40,41,42,43].

**Table 1:** Characteristics of included studies.

Author, Ref.	Country	Study Design	Cancer Site	Sample Size	Female (%)	Exposure	NOS Score
Valdelamar [36]	Colombia	Prospective cohort	Breast, prostate, cervical, and gastrointestinal	400	64.5%	Loneliness	6
Zhao [37]	USA	Retrospective cohort	All	3371	55.4%	Loneliness	8
Beasley [38]	USA	Prospective cohort	Invasive breast	4589	100%	Social isolation	6
Cassileth [39]	USA	Prospective cohort	Advanced pancreatic, gastric, non-small-cell lung cancer, colorectal, and glioma	204	37%	Social isolation	8
Goodwin [40]	USA	Prospective cohort	Breast, prostate, and colon or rectum	646	N/A	Social isolation	9
Kroenke [41]	USA and China	Prospective cohort	Invasive breast	9267	100%	Social isolation	8
Reynolds [42]	USA	Prospective cohort	Breast	1011	100%	Social isolation	9
Sarma [43]	USA	Prospective cohort	Colorectal adenocarcinoma	668	100%	Social isolation	7
Takemura [44]	Japan	Prospective cohort	Advanced lung	211	21%	Loneliness	8

Abbreviations: ref.—reference; NOS—Newcastle–Ottawa scale; N/A—not available.

**Table 2:** Results of sensitivity analyses.

Criterion	Measured Social Connection Construct	Mortality	Statistical Heterogeneity
Excluding NOS < 7	Loneliness	Overall mortality; HR = 1.61 (1.22–2.11), *p* = 0.0006	I^2^ = 0%, Chi^2^ = 0.58, *p* = 0.45
Excluding NOS < 7	Social isolation	Overall mortality HR = 1.36 (1.16–1.60), *p* = 0.0002 Cancer-specific mortality; HR = 1.21 (1.00–1.45), *p* = 0.05	I^2^ = 35%, Chi^2^ = 7.70, *p* = 0.17 I^2^ = 21%, Chi^2^ = 7.62, *p* = 0.27
Excluding different ref. category and unadjusted estimates	Loneliness	Overall mortality; HR = 1.61 (1.22–2.11), *p* = 0.0006	I^2^ = 0%, Chi^2^ = 0.58, *p* = 0.45
Leave one out	Loneliness	Overall mortality; HR range of 1.61–1.87	I^2^ range of 0–45%
Leave one out	Social isolation	Overall mortality; HR range of 1.32–1.44 Cancer-specific mortality; HR range of 1.08–1.34; exclusion of Kroenke; LACE/NHS/SBCSS HR = 1.08 (95% CI 0.89–1.31, *p* = 0.43)	I^2^ range of 0–35% I^2^ range of 0–21%

Abbreviations: NOS—Newcastle–Ottawa scale, HR—hazard ratio.

## Data Availability

The original contributions presented in this study are included in this article/Supplementary Materials.

## Supplementary Materials

The following supporting information can be downloaded at: https://www.mdpi.com/article/10.3390/epidemiologia7050135/s1, Table S1: PRISMA checklist; Table S2: Literature search strategy; Table S3: Exposure assessment and outcomes across included studies.
